# *Abl1* deletion in gut stem cells suppresses p53 induction and promotes colitis-associated tumor formation

**DOI:** 10.1093/jmcb/mjaa022

**Published:** 2020-07-11

**Authors:** Guo Yu, Jie Fu, Ana Romo, Baojie Li, Huijuan Liu

**Affiliations:** 1 Bio-X Institutes, Key Laboratory for the Genetics of Developmental and Neuropsychiatric Disorders, Ministry of Education, Shanghai Jiao Tong University, Shanghai 200240, China; 2 School of Basic Medical Science, Xinxiang Medical University, Xinxiang 453003, China; 3 Laboratory of Stem Cells and Gene Therapy, Instituto Tecnológico de Chascomús (INTECH), CONICET-UNSAM, Chascomús, Buenos Aires, Argentina

**Dear Editor**,

*Abl1*, when fused with *BCR*, expresses fusion protein BCR‒ABL that underlies the etiology of chronic myeloid leukemia and is therapeutically targeted by Imatinib Mesylate (Khatri et al., 2016). However, the function of proto-oncogene product Abl1 remains not fully understood. This non-receptor tyrosine kinase can be activated by growth factors, DNA damage, oxidative stress, and microbial pathogens (Wang, 2014). Cell-based studies suggest that Abl1 phosphorylates proteins in DNA damage response (DDR) and other signaling pathways, promoting p53 expression as well as cell cycle arrest and apoptosis (Gonfloni et al., 2009). *Abl1* deletion leads to runtedness, osteoporosis, and other developmental defects in mice. Interestingly, it has been reported that Abl1 kinase is activated in many solid tumors and Abl1 is implicated in EphB2-mediated intestinal adenoma growth and colorectal cancer (CRC) invasion and metastasis (Kundu et al., 2015; Sonoshita et al., 2015). However, a recent study showed that Abl1 expression is reduced in most CRC patient samples (Uhlen et al., 2015). Thus, the function of Abl1 in CRC initiation warrants further investigation.

**Figure 1:**
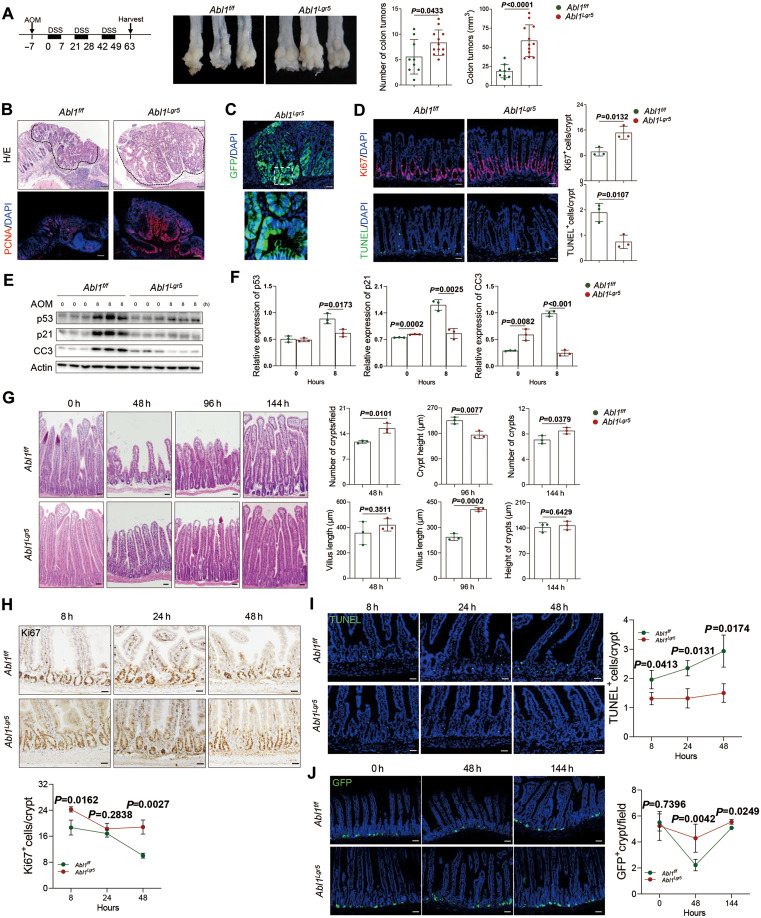
*Abl1* deletion in gut stem cells suppresses DDR and promotes colitis-associated tumor formation. (**A**) *Abl1^Lgr5^* mice show enhanced tumorigenesis in AOM/DSS-induced CAC model. Left: a graph illustrating AOM (10 mg/kg) and DSS (2%) administration. middle: representative images of CAC in *Abl1^f/f^* (*n *=* *9) and *Abl1^Lgr5^* (*n *=* *12) mice. Right: quantitative data. (**B**) H/E and PCNA staining for tumors in *Abl1^f/f^* and *Abl1^Lgr5^* mice. Scale bar, 100 µm. (**C**) GFP staining indicating Lgr5-positive tumor cells. Scale bar, 100 µm. (**D**) *Abl1^Lgr5^* mice show increased proliferation and decreased apoptosis in colorectum in response to AOM treatment. Scale bar, 50 µm. *n *=* *3. (**E**) Western blot results showing that the p53-involved DDR pathway is impaired in *Abl1^Lgr5^* mouse colorectal samples. *n *=* *3. (**F**) Quantitative data for **E**. (**G**) H/E staining revealing that *Abl1^Lgr5^* mice show improved villus regeneration post-IR. Right: quantitative data. *n *=* *3. (**H**‒**J**) Immunostaining revealing that *Abl1^Lgr5^* mice show increased cell proliferation (**H**), decreased apoptosis (**I**), and increased number of Lgr5^+^ ISCs (**J**). *n *=* *3. Quantitative data are shown as mean ± SD.

Here, we tested the possible roles of Abl1 in colitis-associated cancer (CAC). Inflammatory bowel disease is a major driving force of CRC (Choi et al., 2017), which involves genomic instability, inflammation, and oxidative stress, events regulated by Abl1. We deleted *Abl1* in Lgr5^+^ intestinal stem cells (ISCs) by generating *Lgr5-eGFP-CreERT*;*Abl1^f/f^*;*Rosa-tdTomato* mice (Barker et al., 2007). These mice showed Tomato labeling in 60% of crypts one month after three daily doses of tamoxifen (TAM) (Supplementary Figure S1A), suggesting that 60% of Lgr5^+^ ISCs and crypts could have *Abl1* deletion. This was confirmed by quantitative polymerase chain reaction and western blot analyses (Supplementary Figure S2A and G). The mutant (*Abl1^Lgr5^*) mice appeared normal with unaltered colorectal structures (Supplementary Figure S2B). The number and height of crypts and numbers of goblet and Ki67^+^ cells were not significantly altered neither (Supplementary Figure S2B‒D). However, the number of TUNEL^+^ cells was higher in the colorectums of *Abl1^Lgr5^* mice (Supplementary Figure S2E). We checked the p53 pathway and found a negligible increase in p53, p21, and cleaved caspase-3 (CC3) levels in mutant samples (Supplementary Figure S2F). Nevertheless, homeostasis of colorectal crypts is not obviously affected by *Abl1* deletion in ISCs.

We then induced CAC in *Abl1^Lgr5^* mice by azoxymethane/dextran sodium sulfate (AOM/DSS) treatment (Figure 1A). *Abl1^Lgr5^* mice showed increases in the number and size of colorectal tumors (Figure 1A), associated with increased cell proliferation (Figure 1B). Some of the tumor cells were GFP^+^ (Figure 1C), which is driven by the *Lgr5* promoter, suggesting that the tumors are originated from Lgr5^+^ ISCs. These results indicate that *Abl1* deletion in Lgr5^+^ ISCs promotes cell proliferation and tumorigenesis in AOM/DSS-induced CAC model.

CAC development involves microbial metabolites as well as immune and epithelial cells-secreted cytokines and growth factors (Foersch and Neurath, 2014). To determine how Abl1 deficiency promotes CAC, we tested whether *Abl1* deletion affects DSS-induced colitis. Epithelial cells generate the physical barrier and secrete cytokines to regulate immune response during colitis development (Otsuka et al., 2010). However, in DSS-induced colitis model, no difference was observed in body weight, colon length, apparent score, or histological score between the mutant and control mice (Supplementary Figure S3A‒C), suggesting that Abl1 in Lgr5^+^ ISCs and epithelial cells may not play significant roles in colitis development.

Genomic mutations especially in *Trp53* are often observed in IBD patient samples, which is a major driving force of CAC (Foersch and Neurath, 2014). Since CAC model requires genotoxic agent AOM, we determined whether *Abl1* deletion makes a difference in AOM-activated DDR. Indeed, the crypts of *Abl1^Lgr5^* mice showed increased Ki67^+^ cells but decreased TUNEL^+^ cells (Figure 1C), indicating that AOM-induced cell cycle arrest and apoptosis was impaired. We found that *Abl1^Lgr5^* colorectal samples showed decreases in p-p53(S15), p53, p21, and CC3 levels compared with controls (Figure 1E and F; Supplementary Figure S2G), suggesting that Abl1 is required for optimal activation of p53 *in vivo*. However, *Abl1^Lgr5^* samples did not show any alteration in p-ATM, p-H2AX, p-Chk1(S345), or Chk2(T68) levels (Supplementary Figure S2G). Nor did *Abl1* deletion affect activation of the primary pro-proliferation signaling pathways, i.e. ERK, Akt1, and β-catenin (Supplementary Figure S2G). These results suggest that Abl1 regulates tumorigenesis via DNA damage-induced p53.

*Lgr5* also marks small intestine stem cells. Lineage tracing showed that ∼60% villi were marked by Tomato one month after TAM treatment (Supplementary Figure S1B). We found that villus width and length as well as crypt number and height were not significantly affected by *Abl1* deletion (Supplementary Figure S4A and B). While cell proliferation was not affected, the numbers of goblet cells and TUNEL^+^ cells were slightly increased compared with control mice (Supplementary Figure S4C‒E). Overall, these results suggest that Abl1 plays a minor role in intestinal villus turnover.

No tumors were observed in small intestines of *Abl1^Lgr5^* or control mice treated with AOM/DSS (Supplementary Figure S4F). We also used villus to test the roles of *Abl1* in p53 induction, as villus is sensitive to ionizing radiation (IR). We found that after IR (6 Gy), *Abl1^Lgr5^* mouse villi showed better recovery, manifested by greater crypt height, villus length, and crypt number (Figure 1G). Moreover, *Abl1^Lgr5^* mouse intestinal samples showed increases in numbers of proliferating cells and Lgr5^+^ ISCs whereas a decrease in the number of apoptotic cells (Figure 1H‒J).

We conclude that Abl1 acts as a tumor suppressor in CAC development, consistent with decreased Abl1 expression in CRC samples. Abl1 executes its anti-tumor activity likely by regulating p53 expression in response to genotoxic agents but not inflammation.

*[Supplementary material is available at Journal of Molecular Cell Biology online. The work was supported by the National Key Research and Development Program of China (2017YFA0103602 and 2018YFA0800803) and the National Natural Science Foundation of China (81520108012 and 91749201).]*

## Supplementary Material

mjaa022_Supplementary_Material
