# Supplementary material for: Abl1 deletion in gut stem cells suppresses p53 induction and promotes colitis-associated tumor formation
Source: J Mol Cell Biol. 2020 Jul 11;12(9):738–40. doi: 10.1093/jmcb/mjaa022 (PMC7749737; doi:10.1093/jmcb/mjaa022)
Supplement: mjaa022_Supplementary_Material [file mjaa022_supplementary_material.pdf]

Supplementary material

Abl1 deletion in gut stem cells suppresses p53 induction and promotes colitis-associated tumor formation

Guo Yu[1], Jie Fu[2], Ana Romo[3], Baojie Li[1], and Huijuan Liu[1,*]

[1]Bio-X Institutes, Key Laboratory for the Genetics of Developmental and Neuropsychiatric Disorders, Ministry of Education, Shanghai Jiao Tong University, Shanghai 200240, China

[2]School of Basic Medical Science, Xinxiang Medical University, Xinxiang 453003, China

[3]Laboratory of Stem Cells and Gene Therapy, Instituto Tecnológico de Chascomús (INTECH), CONICET-UNSAM, Chascomús, Buenos Aires, Argentina

* Correspondence to: Huijuan Liu, E-mail: liuhj@sjtu.edu.cn

Materials and methods

Mice. *Rosa-tdTomato* and *Lgr5-eGFP-CreERT* (*Lgr5*^{eGFP}) mouse lines were purchased from The Jackson Laboratory and *Abl1*$^{f/f}$ mice were provided by Dr. Stephen Goff at Columbia University. These mice were maintained at the SPF room of the Bio-X Institutes, Shanghai Jiao Tong University. For all experiments 8–10-weeks old gender-matched mice were used. Littermates carrying loxP-flanked alleles without Cre served as control mice. Mice were randomly assigned to the groups and the studies were performed in a blinded fashion.

CAC model generation. AOM was dissolved in sterile PBS and injected intraperitoneally at a dose of 10 mg/kg. Two percent DSS was provided in drinking water. Mice were treated for 3 cycles (Neufert et al., 2007). Animals were sacrificed after 10 weeks. Tumor volume was calculated according to the following formula: volume = ($L \times l^2 / 2$), in which L represents the longest diameter and l represents the shortest diameter.

Histology and immunohistochemistry. Samples were fixed overnight in 4%

paraformaldehyde, embedded in paraffin and cut into 5 μm sections. Paraffin sections were rehydrated and heat-induced antigen retrieval was performed in 10 mM Sodium Citrate, 0.05% Tween-20 at pH 6. Primary antibodies were Ki67 (Thermo Scientific; PA5-19462; 1:100 dilution), PCNA (PC10) (Cell Signaling, 2586; 1:100 dilution), GFP (D5.1) (Cell Signaling; 2956; 1:100 dilution). For IHC, biotinylated secondary antibodies were purchased from Boster Biological Technology. Staining was visualized with ABC Kit (BOSTER). For fluorescent staining, Alexa Fluor (Invitrogen) fluorescent secondary antibodies were used. Cell death was evaluated using *in situ* cell death detection kit (Roche). Pictures were taken with a fluorescence microscope (Olympus) under the same exposure and intensity settings.

Immunoblotting. Total proteins extracted from colon and small intestine were separated by SDS–polyacrylamide electrophoresis gels and transferred onto Immobilon-polyvinylidene difluoride membranes (Millipore). Membranes were incubated with primary antibodies; anti-Abl1 (Santa Cruz Biotechnology; sc-131; 1:1,000 dilution), anti-p53 (1C12) (Cell Signaling; 2524; 1:1,000 dilution), anti-p21(187) (Santa Cruz Biotechnology; sc-817; 1:1,000 dilution), anti-cleaved caspase-3 (D175) (Cell Signaling; 9661; 1:1,000 dilution), anti-β-Catenin (H-102) (Santa Cruz Biotechnology; sc-7199; 1:1,000 dilution), anti-p-Akt (S473)(D9E)XP(R) (Cell Signaling; 4060; 1:1,000 dilution), anti-Akt (Cell Signaling; 9272; 1:1,000 dilution), anti-p-Erks (Cell Signaling; 4376; 1:1,000 dilution), anti-Erks (Cell Signaling; 9107; 1:1,000 dilution), anti-p-Chk1 (S345) (Cell Signaling; 2348; 1:1,000 dilution), anti-Chk1 (Cell Signaling; 2360; 1:1,000 dilution), anti-p-Chk2 (T68) (Cell Signaling; 2661; 1:1,000 dilution), anti-Chk2 (Cell Signaling; 2662; 1:1,000 dilution), anti-p-p53 (S15) (Cell Signaling; 9284; 1:1,000 dilution), anti-p-ATM(Cell Signaling; 4526; 1:1,000 dilution) and anti-actin (Santa Cruz Biotechnology; sc-47778; 1:3,000 dilution) antibodies at 4 ℃ overnight. Membranes were incubated with secondary horseradish peroxidase antibodies (Invitrogen) and developed with chemiluminescent detection substrate (Thermo Scientific).

Irradiation experiments. For ionizing radiation experiments, mice were irradiated using Rad Source RS2000 machine and time was set for 360 seconds (the dose for 60

seconds was 1Gy) (He et al., 2020).

TUNEL assay. Samples were fixed overnight in 4% paraformaldehyde, embedded in paraffin and cut into 5 μm sections. Paraffin sections were rehydrated and were pretreated with proteinase K at 37 ℃ for 10 mins. Then place slides in 10 mM Sodium Citrate, 0.05% Tween-20 at pH 6 for 15 mins. adding 50 μl TUNEL reaction mixture (Roche, *In Situ* Cell Death Detection Kit) on samples and incubating at 37 ℃ for 60 mins. Samples were embedded with antifade prior to analysis.

Quantitative PCR. Total RNA was extracted from colorectum using Trizol (Invitrogen). cDNA was synthesized with random primers and amplified in duplicate by QuantiTect SYBR Green PCR kit (Roche) using an ABI 7500 sequence detector (Applied Biosystems). Primer sequences were as following:

Abl1-forward: CAG CAG CCT GGA AAA GTT CTT

Abl1-reversed: CCC TGC CCC TTT GAT AAA ATG C

Actin-forward: ACA GCT TCT TTG CAG CTC CTT

Actin-reversed: TGG TAA CAA TGC CAT GTT CAA T

Statistical analysis. Data were subjected to Student ́s t-test using GraphPad Prism software. Results are shown as mean ±s.d. Groups were large enough to fulfil the prerequisites of Student's *t*-test as well as to determine whether the variance between groups is not significantly different.

Author contributions

G.Y., A.R., and J.F. performed experiments and B.L. and H.L. designed experiments and wrote the paper.

References

He, D., Wu, H., Xiang, J., et al. (2020). Gut stem cell aging is driven by mTORC1 via a p38 MAPK-p53 pathway. Nat. Commun. *11*, 37.

Neufert, C., Becker, C., and Neurath, M.F. (2007). An inducible mouse model of colon carcinogenesis for the analysis of sporadic and inflammation-driven tumor progression. Nat. Protoc. *2*, 1998-2004.

Supplementary Figures

Supplementary Figure S1

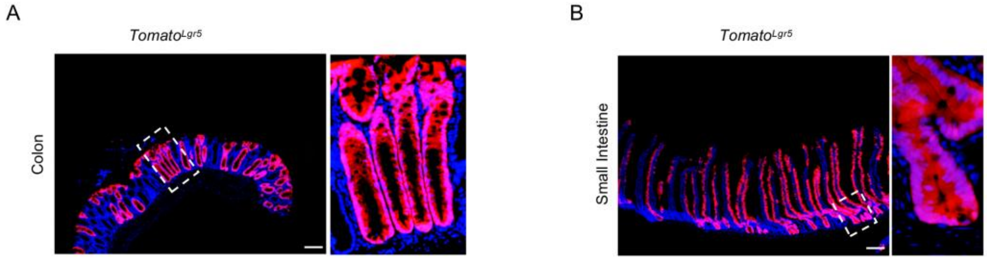

Supplemenary Figure S1 Lineage tracing of Lgr5^{+} ISCs and their progenies in colorectum (**A**) and small intestine (**B**). The mice were sacrificed one month after three doses of 10 mg/kg tamoxifen. Scale bar, 200 μm.

Supplementary Figure S2

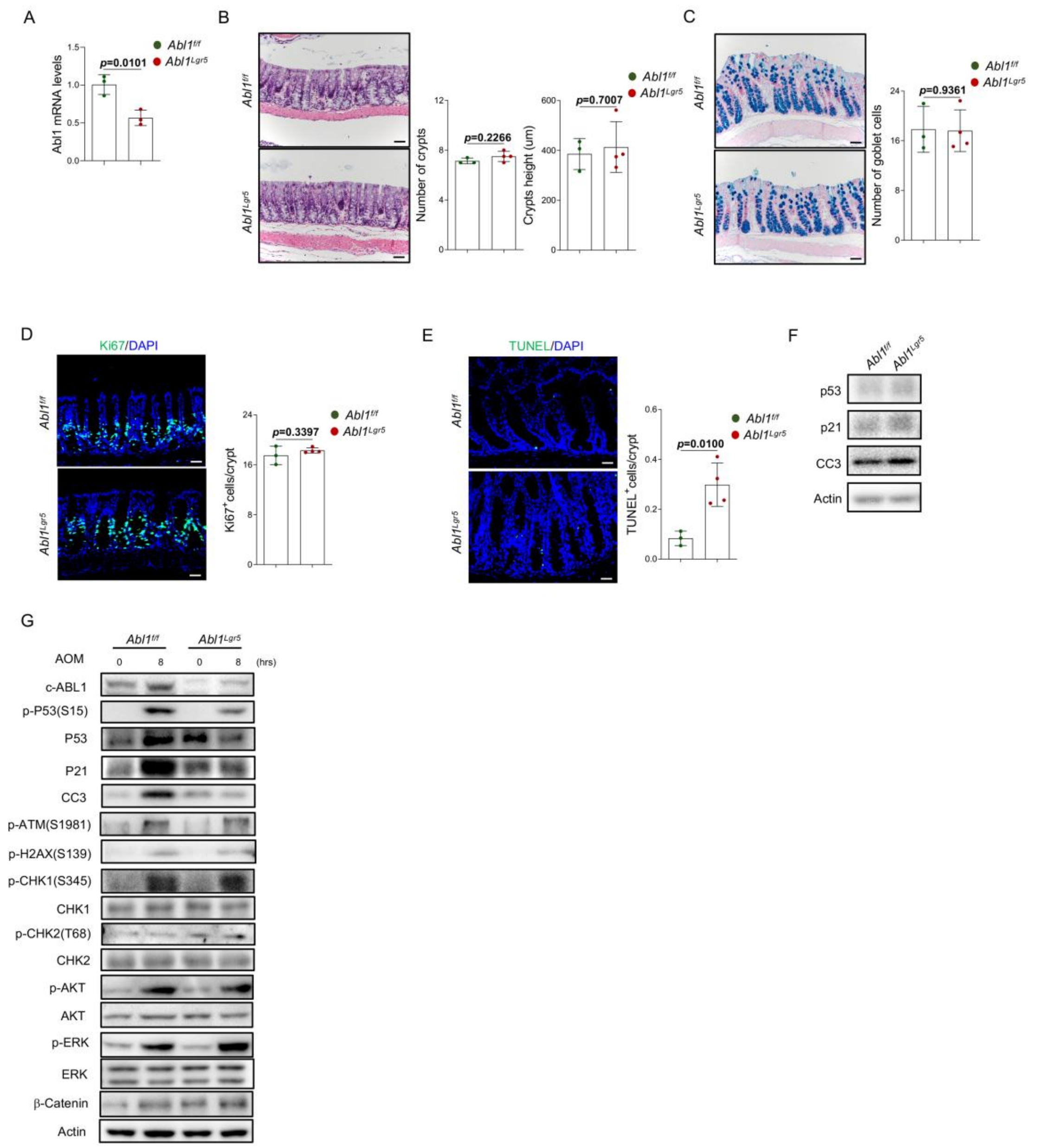

Supplemenary Figure S2 Homeostasis of colorectum is not affected in *Abl1*^{Lgr5} mice. (**A**) Quantitative PCR results showing that Abl1 mRNA levels are reduced in colorectal samples of *Abl1*^{Lgr5} mice. (**B**) Representative images of H/E-stained colon sections of *Abl1*^{Lgr5} and control mice. Right: quantitative data. *n*=3. (**C**) Representative images of goblet cell staining and quantitative data. *n*=3. (**D**) Representative images of Ki67 staining and quantitative data. *n*=3. (**E**) Representative images of TUNEL staining and quantitative data. *n*=3. (**F**) Immunoblotting analysis of colorectal samples for p53, p21, and CC3. (**G**) Immunoblotting analysis of colorectal

samples after one dose of AOM injection for Abl1, p-ATM, p-H2AX, p-Chk1, p-Chk2, p-p53, p-p53, p21, p-ERK, p-Akt1, and β-catenin. For all experiments, data are shown as mean ±SD. Scale bar, 50 μm.

Supplementary Figure S3

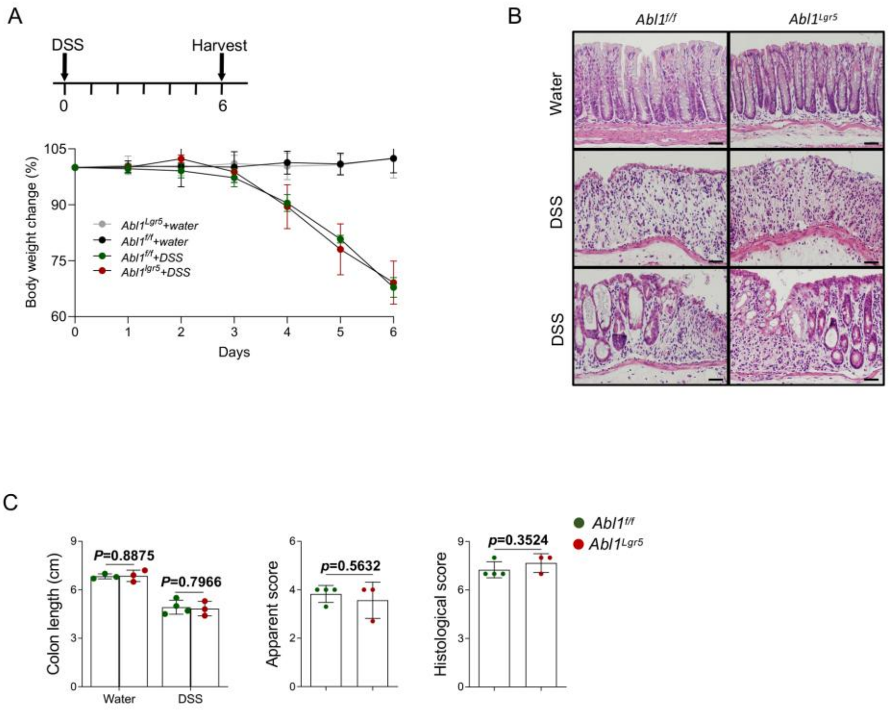

Supplemenary Figure S3 *Abl1* deletion in Lgr5$^+$ ISCs does not affect DSS-induced colitis. (**A**) *Abl1*^{Lgr5} mice treated with 3% DSS show similar decreases in body weight as control mice. *n*=4 for *Abl1*$^{f/f}$ mice and *n*=3 for *Abl1*^{Lgr5} mice. (**B**) Representative images showing that *Abl1*^{Lgr5} mice display similar structure deterioration as control mice. Scale bar, 50 μm. (**C**) *Abl1*^{Lgr5} mice showed similar changes in colon length, apparent score, and immunohistochemical score as control mice. *n*=4 for *Abl1*$^{f/f}$ mice and *n*=3 for *Abl1*^{Lgr5} mice.

Supplementary Figure S4

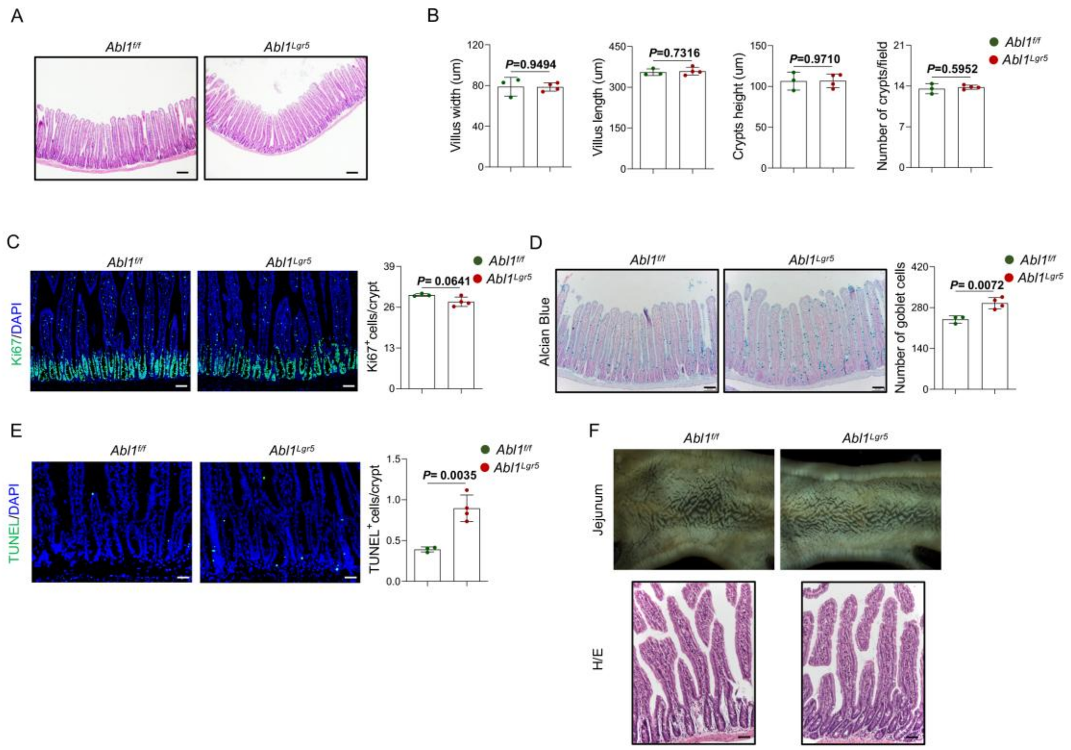

Supplemenary Figure S4 Homeostasis of small intestinal villi is normal in *Abl1*^{Lgr5} mice. (**A**) Representative images showing that *Abl1*^{Lgr5} mice display similar small intestinal structures as control mice. Scale bar, 200 μm. (**B**) Quantitative data for crypt number and height as well as villus width and length. *n*=3. (**C**) Representative images of Ki67 staining on small intestine sections of *Abl1*^{Lgr5} and control mice. Right: quantitative data. *n*=3. Scale bar, 100 μm. (**D**) Representative images of goblet cell staining on small intestine sections of *Abl1*^{Lgr5} and control mice. Right: quantitative data. *n*=3. Scale bar, 100 μm. (**E**) Representative images of TUNEL staining on small intestine sections of *Abl1*^{Lgr5} and control mice. Right: quantitative data. *n*=3. Scale bar, 50 μm. (**F**) Representative images of small intestine and H/E stained sections of *Abl1*^{Lgr5} and control mice following AOM/DSS treatment. Scale bar, 50 μm. For all experiments, data are shown as mean ±SD.
